# Nitrite Reductase Activity and Inhibition of H_2_S Biogenesis by Human Cystathionine ß-Synthase

**DOI:** 10.1371/journal.pone.0085544

**Published:** 2014-01-08

**Authors:** Carmen Gherasim, Pramod K. Yadav, Omer Kabil, Wei-Ning Niu, Ruma Banerjee

**Affiliations:** 1 Department of Biological Chemistry, University of Michigan Medical School, Ann Arbor, Michigan, United States of America; 2 School of Life Science, Northwestern Polytechnical University, Xi’an, China; Texas A&M University, United States of America

## Abstract

Nitrite was recognized as a potent vasodilator >130 years and has more recently emerged as an endogenous signaling molecule and modulator of gene expression. Understanding the molecular mechanisms that regulate nitrite metabolism is essential for its use as a potential diagnostic marker as well as therapeutic agent for cardiovascular diseases. In this study, we have identified human cystathionine ß-synthase (CBS) as a new player in nitrite reduction with implications for the nitrite-dependent control of H_2_S production. This novel activity of CBS exploits the catalytic property of its unusual heme cofactor to reduce nitrite and generate NO. Evidence for the possible physiological relevance of this reaction is provided by the formation of ferrous-nitrosyl (Fe^II^-NO) CBS in the presence of NADPH, the human diflavin methionine synthase reductase (MSR) and nitrite. Formation of Fe^II^-NO CBS via its nitrite reductase activity inhibits CBS, providing an avenue for regulating biogenesis of H_2_S and cysteine, the limiting reagent for synthesis of glutathione, a major antioxidant. Our results also suggest a possible role for CBS in intracellular NO biogenesis particularly under hypoxic conditions. The participation of a regulatory heme cofactor in CBS in nitrite reduction is unexpected and expands the repertoire of proteins that can liberate NO from the intracellular nitrite pool. Our results reveal a potential molecular mechanism for cross-talk between nitrite, NO and H_2_S biology.

## Introduction

NO regulates a wide range of physiological processes including vasorelaxation, neurotransmission and immune responses [1]–[3]. Its potency as a signaling molecule relies on its short half-life, limited diffusibility and high reactivity with heme proteins [4]. The primary source of NO is nitric oxide synthase (NOS), which oxidizes L-arginine to generate L-citrulline and NO. The discovery of NO-mediated hypoxic vasorelaxation suggested the presence of additional sources of NO under oxygen-limiting conditions where NOS is inactive [5]. Biochemical and physiological evidence exists suggesting that nitrite, the one electron oxidation product of NO, represents a circulating pool of NO that can be accessed under hypoxic conditions [6]. In addition to nonenzymatic acidic reduction of nitrite, enzyme-mediated nitrite reduction has also been reported [7], [8]. To date, a limited number of hemeproteins, such as globins and cytochrome *c* have been identified as nitrite reductases albeit their activities cannot entirely account for the positive effects of nitrite treatments [5], [9]–[12]. Furthermore, clinical studies that have investigated the usefulness of nitrite as an NO donor identified a non-linear dose-dependent increase of nitrite concentration with administered nitrite suggesting additional players for nitrite clearance [13], [14].

Human CBS is a 5′-pyridoxal phosphate (PLP)- and heme-containing protein that controls the levels of key sulfur metabolites including homocysteine, glutathione and H_2_S [15]–[17]. Genetic defects in CBS represent the most common cause of hereditary homocystinuria, an inborn error of metabolism associated with aggressive occlusive arterial disease [18]. CBS uses its PLP cofactor to catalyze ß–replacement reactions that contribute to homocysteine clearance in the presence of either serine or cysteine as a co-substrate. The ß-replacement of serine with homocysteine represents the cannonical reaction in the transsulfuration pathway, while the ß-replacement of cysteine with homocysteine results in H_2_S biogenesis [19], [20]. A unique heme b cofactor in human CBS represents a puzzling evolutionarily accessory whose role is unclear [21]. While the heme is not required for enzyme activity, both structural and regulatory roles have been proposed for it [22]–[24]. Binding of carbon monoxide (CO) or NO to the Fe^II^-CBS heme inhibits enzyme activity [25], [26]. We have demonstrated that despite the low reduction potential (−350 mV) for the Fe^3+^/Fe^2+^ couple of CBS [27], reversible regulation by CO binding can be achieved with physiologically relevant reductants like methionine synthase reductase (MSR) and novel reductase 1 [24], [28].

The growing interest in H_2_S signaling, which mediates profound physiological effects [29], [30] has focused attention on the enzymes responsible for its biogenesis and decay [31]–[33]. In addition to CBS, the enzymes involved in H_2_S production include cystathionase [34], [35] and mercaptopyruvate sulfurtransferase [36], [37]. Rapid turnover of H_2_S contributes to maintaining its low steady-state concentrations estimated to be in the ∼10–30 nM range [38], [39]. Hence, regulation of both H_2_S production and catabolism are important targets for cellular and pharmacological modulation of its levels [40]. Increasingly, there is evidence for interactions between the H_2_S and the gas-signaling pathways elicited by CO and NO but the molecular mechanisms for this cross-talk are poorly understood [41].

Changes in the heme ligation or spin state are conveyed over a long distance to the active site of CBS and inhibits its PLP-dependent activity [26], [42]. The ability of flavin oxidoreductases to generate Fe^II^-CBS in the presence of NADPH [24] suggested that the allosteric heme sensor domain might exhibit additional regulatory strategies. Herein, we report a previously unknown function of the heme in human CBS, i.e. reaction with nitrite to form Fe^II^-NO, which inhibits H_2_S formation. Our results suggest a possible molecular mechanism for crossover between the NO and H_2_S signaling pathways.

## Results

### Nitrite Reductase Activity of CBS

**Figure 1:**
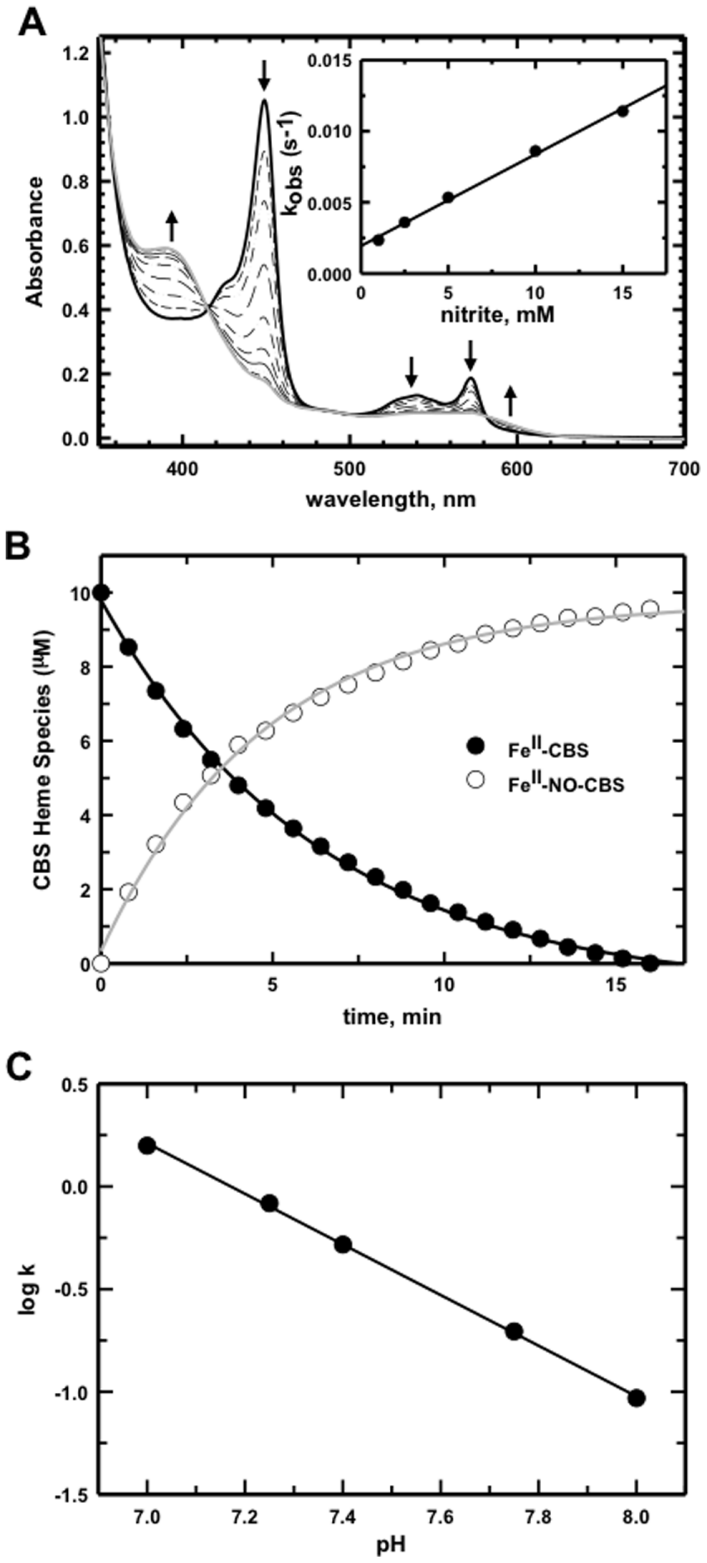
Nitrite reduction by Fe^II^-CBS. (**A**) UV-visible spectra recorded every minute under anaerobic conditions for the reaction between Fe^II^-CBS (10 µM, generated by reduction of Fe^III^-CBS with 3 mM dithionite) and nitrite (10 mM) in 0.1 M HEPES buffer, pH 7.4, at 37°C. (*Inset*) The observed reaction rate for CBS-catalyzed reaction as a function of nitrite concentration. (**B**) The disappearance of Fe^II^-CBS (formed by reduction of Fe^III^-CBS (10 µM) with dithionite (3 mM)) was monitored at 449 nm (filled circles) and paralleled the formation of Fe^II^-NO-CBS in the presence of 10 mM nitrite (open circles) monitored at 394 nm. The solid lines represent single exponential fits to the experimental data points. (**C**) Dependence of nitrite reduction by Fe^II^-CBS on pH. Reaction of Fe^II^-CBS (10 µM) generated by the reduction of Fe^III^-CBS with dithionite (3 mM) in 0.1 M HEPES pH 7.0, 7.25, 7.4, 7.75 and 8.0 at 37°C with nitrite (10 mM) was monitored at 449 nm. Reaction rates corrected for the percentage of reduced protein at each pH were plotted as a function of pH. The slope obtained from a linear fit was 1.2±0.03.

The release of NO from nitrite is mediated by interactions between Fe^II^-hemoglobins and nitrite [5], [9], [10] and the possible involvement of other heme proteins in this process is implicated [11]. Long-term sodium nitrite administration displayed a non-linear increase in nitrite concentrations, which was modeled as an increase in its clearance [14]. We therefore examined the interaction of Fe^II^-CBS with nitrite. Reaction of 10 µM Fe^II^-CBS with 10 mM nitrite under anaerobic conditions, showed time- and nitrite concentration-dependent changes in the heme spectrum consistent with formation of Fe^II^-NO CBS (Fig.1A). The decrease in absorbance of Fe^II^-CBS with a Soret maximum at 450 nm and α and ß bands at 540 and 570 nm, respectively was accompanied by the appearance of a 394 nm peak and broadening of the α/β bands, corresponding to formation of five-coordinate Fe^II^-NO CBS. The data for Fe^II^-CBS disappearance and Fe^II^-NO CBS formation were fitted to single exponential functions, and yielded *k*_obs_ = 0.52 min^−1^ (Fig. 1B). In analogy to globins [46]–[48], the mechanism for nitrite reduction by CBS is summarized by equations 1 and 2.
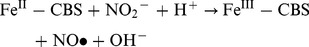
(1)

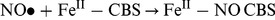
(2)


The kinetics of the nitrite reduction reaction in the presence of excess sodium dithionite is consistent with one Fe^II^-CBS forming one Fe^II^-NO CBS (Fig. 1B). Furthermore, the isosbestic conversion of the ferrous species to Fe^II^-NO indicates that Fe^III^-CBS does not accumulate, i.e. it is rapidly reduced to Fe^II^-CBS, which reacts with NO. The bimolecular rate constant calculated from the linear fit of *k*_obs_ as a function of nitrite concentration is 0.6 M^−1^ s^−1^ at 37°C, pH 7.4 (Fig. 1A, *inset*). In the presence of the allosteric activator of CBS, S-adenosylmethionine, the nitrite reductase activity was increased ∼2-fold (*k*_obs_ = 0.98 min^−1^).

Since the Fe^II^-CBS-dependent nitrite reduction is predicted to require a proton (equation 1), its pH-dependence was studied. A 10-fold increase in the rate of the reaction was observed between pH 8 and 7 (Fig. 1C). The slope of the rate dependence on proton concentration was 1.2±0.03 consistent with the requirement for one proton per Fe^II^-NO CBS formation.

### Nitrite Reduction by CBS in the Presence of a Physiological Reductant

**Figure 2:**
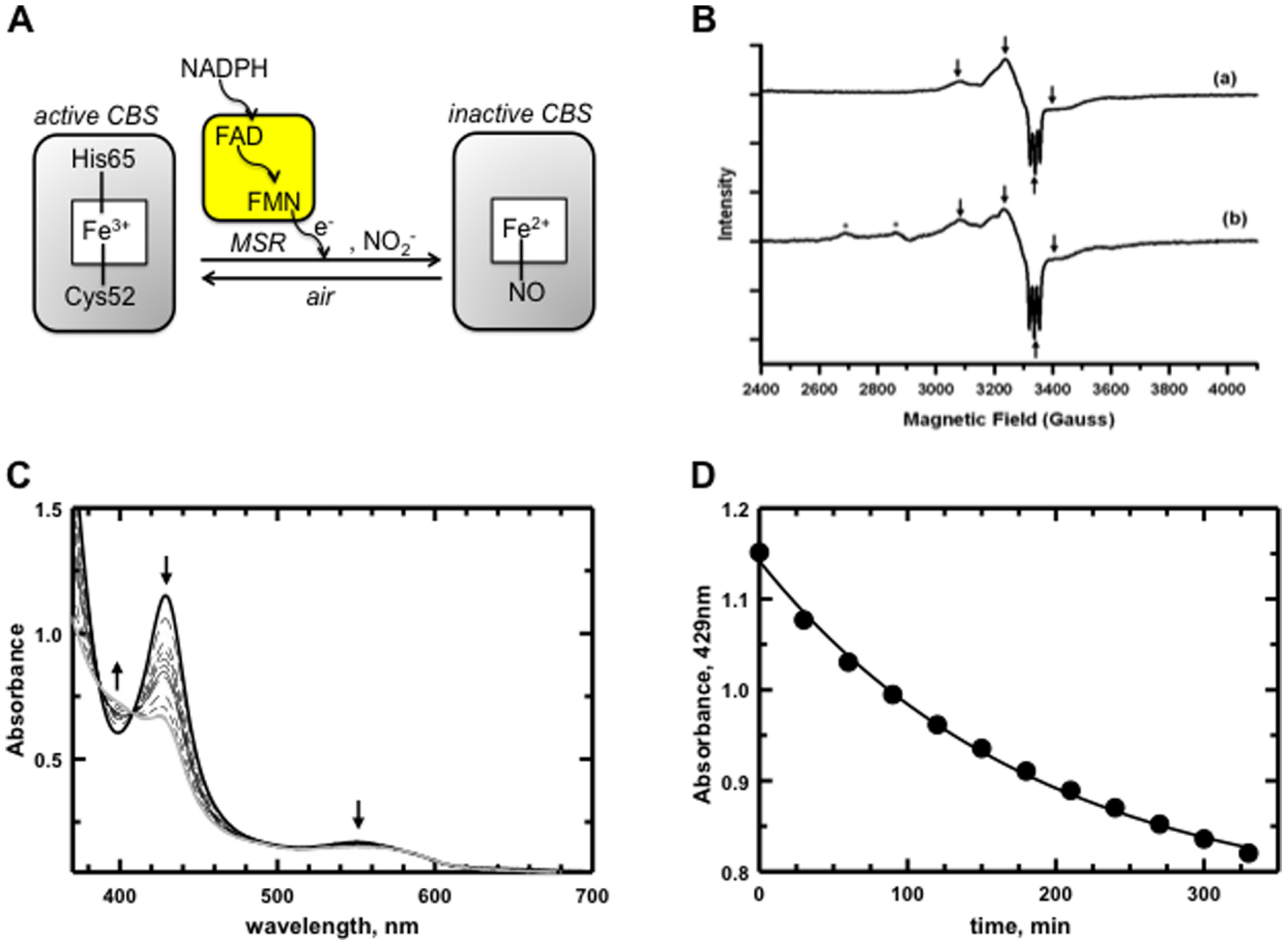
Model for and spectroscopic evidence of formation of Fe^II^-NO CBS in the presence of MSR/NADPH. (**A**) Fe^III^-CBS catalyzes the condensation of cysteine (Cys) and homocysteine (Hcy) to give H_2_S and cystathionine (Cyst). The latter is subsequently cleaved to give cysteine, which is utilized for glutathione (GSH) synthesis. In the presence of NADPH/MSR and nitrite, Fe^II^-NO CBS is formed, rendering CBS inactive. (**B**) EPR spectra of Fe^II^-NO CBS, obtained with Fe^III^-CBS (65 µM), treated with dithionite (6 mM) (upper) or NADPH (2 mM)/MSR (20 µM) (lower) and sodium nitrite (10 mM) in 0.1 M HEPES buffer, pH 7.4 at 37°C. The spectra were recorded using the conditions described previously [26]. The arrows indicate *g* values of 2.17, 2.076, 2.008 and 1.97, respectively. The presence of additional EPR signals in the spectrum of NADPH/MSR-dependent CBS-catalyzed nitrite reduction can be attributed to the incomplete reduction of paramagnetic Fe^III^-CBS. (C) UV-visible spectra were recorded every 10 min under anaerobic conditions for the reaction between Fe^II^-CBS (generated by reduction of Fe^III^-CBS (10 µM) with MSR (2 µM)/NADPH (1 mM)) and nitrite (10 mM) in 0.1 M HEPES buffer, pH 7.4, at 37°C. (B) Time-dependent conversion of Fe^III^-CBS (429 nm) to Fe^II^-NO-CBS (394 nm).

The nitrite reductase activity of CBS in vivo would be contingent upon the presence of a reducing system that generates the reactive Fe^II^ species. MSR can shuttle an electron from NADPH through its flavin cofactors to Fe^III^-CBS [24] (Fig. 2A). In the presence of NADPH, nitrite and substoichiometric MSR, the conversion of Fe^III^- to Fe^II^-NO CBS was observed indicating that nitrite reduction can be achieved in the presence of a biochemical reducing system (Figs. 2B,C). We note that the UV-visible spectrum of the Fe^II^-NO-CBS in this reaction mixture is partially obscured by NADPH oxidation during this experiment. EPR spectroscopy provides further evidence for the formation of the Fe^II^-NO-CBS product as discussed below (Fig. 2B). The observed rate for NADPH/MSR-dependent Fe^II^-NO CBS formation as monitored by the decrease in absorbance at 429 nm is 0.007 min^−1^ (Fig. 2D). The latter is slower than the rate obtained in the presence of dithionite, a more efficient artificial reductant for CBS whose kinetic characterization has been reported by Carballal et al. The results suggest a shift in the rate-limiting step from nitrite reduction in the presence of dithionite to the reduction of CBS-Fe^III^-to Fe^II^ by NADPH/MSR.

### EPR Spectrum of CBS during Nitrite Reduction

The heme in human CBS is six-coordinate in both the Fe^II^ and Fe^III^ states, and Cys52 and His65 serve as axial ligands [22], [49]–[51] (Fig. 2A). The EPR spectrum obtained during CBS-catalyzed nitrite reduction in the presence of dithionite or NADPH/MSR, provides evidence for the formation of paramagnetic, five-coordinate Fe^II^-NO CBS with a characteristic three-line hyperfine splitting resulting from the interaction between the unpaired electron and the *I* = 1 nucleus of the nitrogen in NO (Fig. 2B). Formation of Fe^II^-NO CBS leads to loss of both endogenous ligands [26], unlike other six-coordinate hemeproteins where NO binding displaces only one of the endogenous axial ligands.

### Nitrite Reductase Activity at the Heme Site of CBS Inhibits β-replacement Activity and H_2_S Biogenesis at the PLP Site

**Figure 3:**
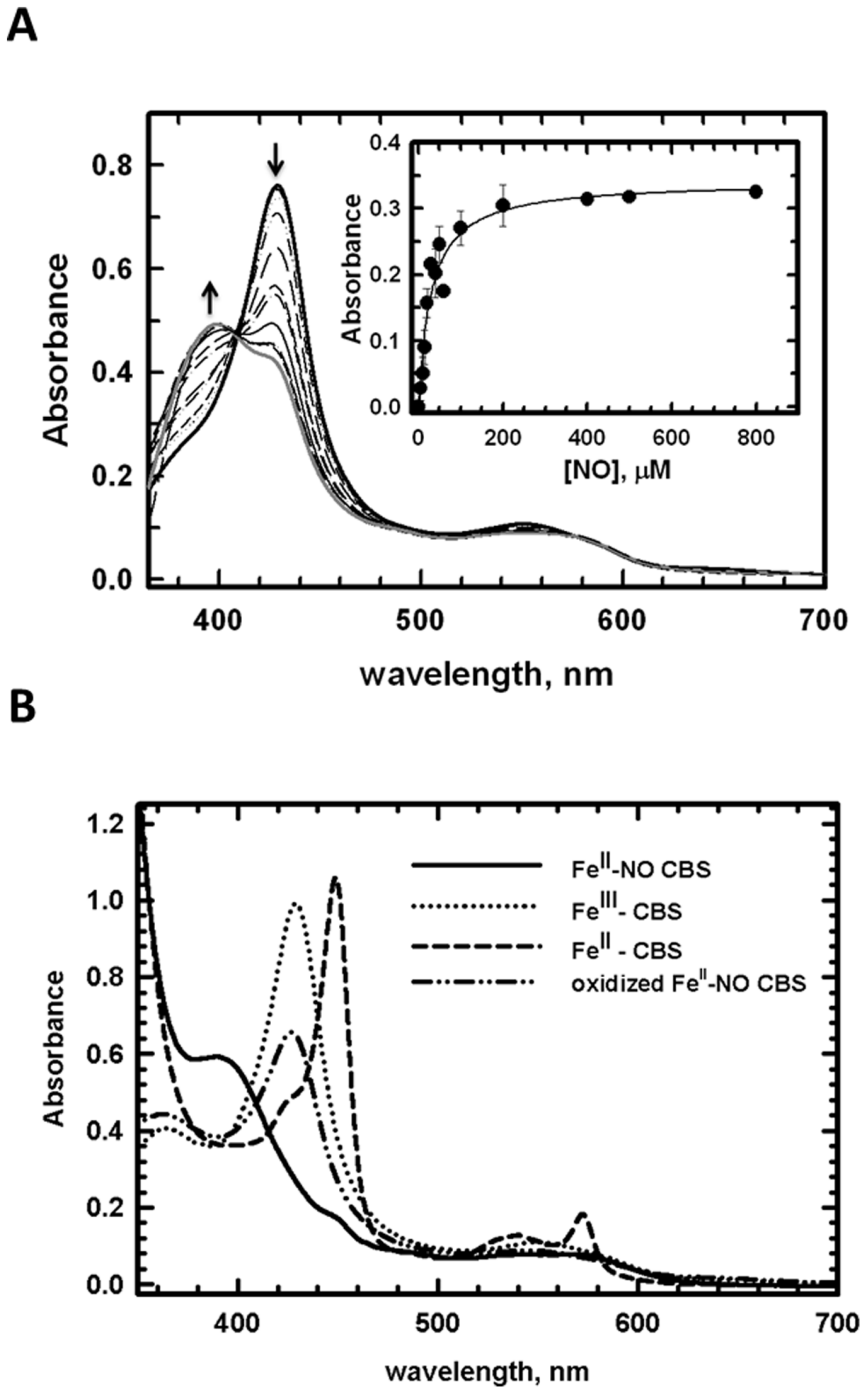
Spectral analysis of reversible NO binding to CBS. (**A**) An anaerobic solution of CBS (10 µM) in 0.1 M HEPES, pH 7.4, was mixed with 250 µM NADPH and 5 µM MSR and varying concentrations (0–533 µM) of diethylamine NONOate in 10 mM NaOH. A sample containing 5 µM MSR and 250 µM NADPH was used as a blank to clarify the region of the spectrum between 350–400 nm. The inset shows the change in absorbance at 428 nm as a function of NO concentration. (**B**) Reversible generation of Fe^II^-NO-CBS by nitrite reduction. Reduction of Fe^III^-CBS (10 µM) (**^….^**) with dithionite (3 mM) yields Fe^II^-CBS (–). Reaction of the Fe^II^-CBS form with nitrite yields Fe^II^-NO-CBS (–), which can be re-oxidized to Fe^III^-CBS (−.−).

Binding of NO to Fe^II^-CBS inhibits its activity in the canonical reaction that generates cystathionine from homocysteine and serine [26]. The K_D_ for NO binding to CBS (281±50 µM) [26] was previously determined at pH 8.6, the pH maximum for CBS activity. However, the presence of excess dithionite (1.5 mM) in the reaction mixture could have resulted in slow reduction of NO as reported previously [52], leading to an overestimation of the K_D_ value for NO binding to CBS. We therefore reassessed binding of NO to CBS at physiological pH (7.4) and employed NADPH and MSR as a source of electrons (Fig. 3A). In the presence of the NO precursor, diethylamine NONOate, a shift in the Soret maximum from 428 nm (corresponding to ferric CBS) to 395 nm (Fe^II^-NO CBS) was observed, consistent with the conversion of six-coordinate low-spin Fe^III^ to five-coordinate high-spin Fe^II^-NO CBS as seen previously [26]. Based on this analysis, a K_Dapp_ for binding of NO to CBS was estimated to be 30±5 µM. We note that this is an apparent K_D_ and represents an *upper* limit, since formation of Fe^II^-NO CBS under these conditions involves multiple equilibria including NADPH binding to MSR, MSR binding to CBS, reduction of CBS by MSR and NO binding to Fe^II^-CBS.

**Figure 4:**
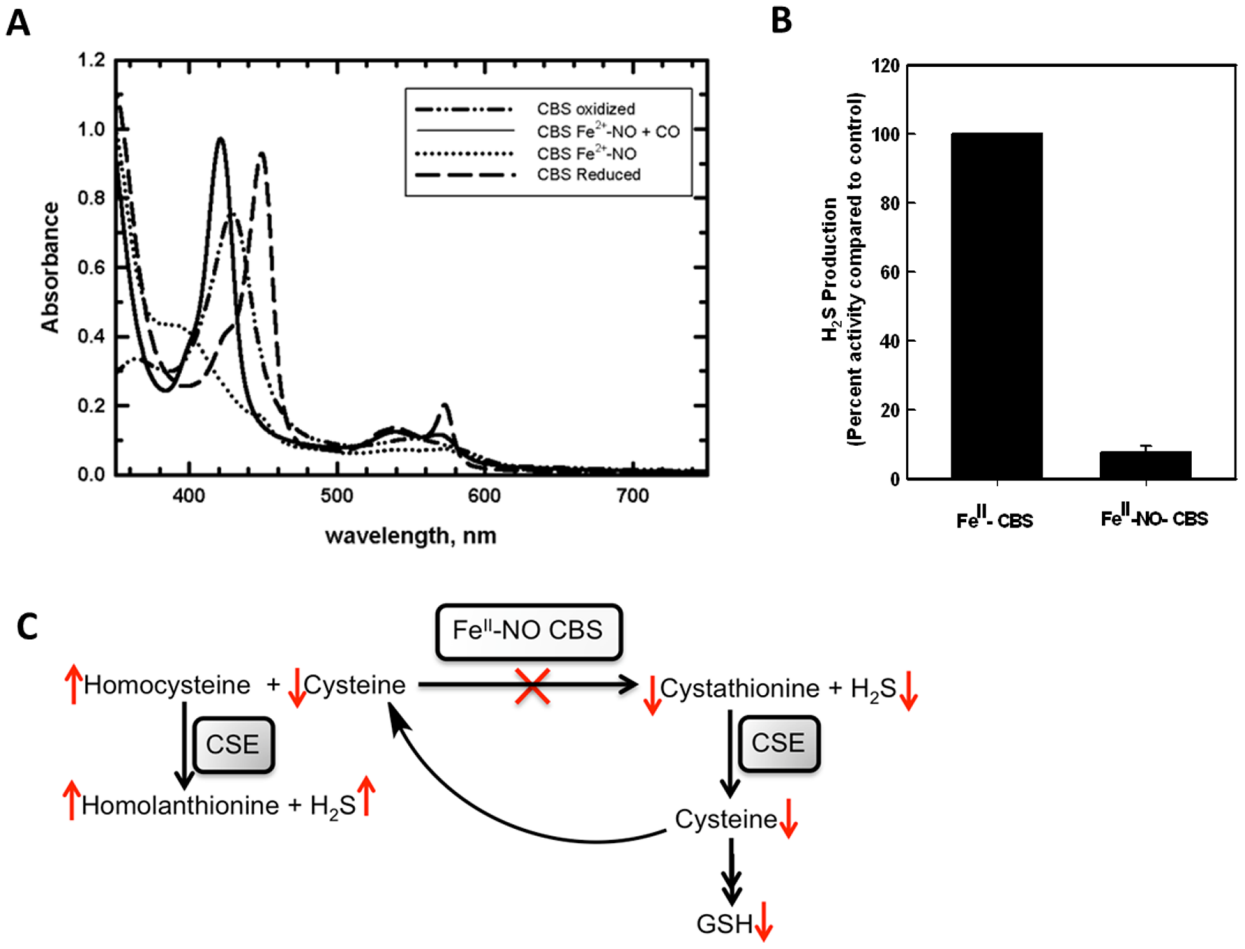
Reversible generation and metabolic consequences of Fe^II^-NO CBS. (**A**) Reduction of Fe^III^-CBS (10 µM) in 0.1 M HEPES buffer, pH 7.4, (-••-) with dithionite (3 mM) yields Fe^II^-CBS (–). The latter reacts with 10 mM nitrite to give Fe^II^-NO CBS (^….^). The NO ligand is exchanged for CO upon incubation of the reaction mixture for 10–15 min with CO (––). (**B**) Effect of NO binding to Fe^II^-CBS on H_2_S production was measured in 0.1 M HEPES buffer, pH 7.4 using cysteine (10 mM) and homocysteine (10 mM) as substrates. H_2_S generation was assesed using the lead sulfide precipitation assay. (**C**) Predicted metabolic consequences of Fe^II^-NO CBS formation. Inhibition of CBS by its nitrite reductase activity is predicted to decrease CBS-dependent H_2_S formation while increasing cystathionase (CSE)-dependent H_2_S formation due to homocysteine accumulation. The concentration of the antioxidant glutathione (GSH), is also predicted to decrease.

Formation of Fe^II^-NO inhibits CBS activity in the canonical serine+homocysteine reaction (82±20 µmol mg^−1^ h^−1^ for Fe^II^-NO CBS versus 257±25 µmol mg^−1^ h^−1^ for Fe^III^-CBS). The reversibility of inhibition by the Fe^II^-NO species was assessed by air-oxidation, which led to the ready formation of Fe^III^-CBS (Fig. 3B). The latter in turn, was accompanied by recovery of activity (195±10 µmol mg^−1^ h^−1^). The incomplete overall recovery of Fe^III^-CBS activity from Fe^II^-NO CBS might be due to nitrite-induced degradation of the heme in air as also reported for human hemoglogin [53]. Partial loss of the heme during the re-oxidation process has been also observed with Fe^II^-CO CBS [24]. The mechanism and physiological role of nitrite-induced heme degradation in air for CBS is presently unclear. While the rate of oxidation of Fe^II^-NO CBS has not been reported yet, oxidation of Fe^II^-CBS occurs rapidly with a second-order rate constant of 1.1×10^5^ M^−1^ s^−1^ (at 25°C and pH 7.4). Assuming similar oxidation kinetics for Fe^II^-NO CBS, we propose that nitrite reduction by CBS can modulate its activity via reversible formation of Fe^II^-NO heme. Displacement of the NO ligand by CO was observed upon incubating Fe^II^-NO CBS with CO, indicating integrity of the heme in the Fe^II^-state (Fig. 4A). In addition to the production of cystathionine in the canonical reaction, CBS generates H_2_S using alternative substrates such as cysteine or cysteine+homocysteine. Formation of Fe^II^-NO CBS was correlated with ∼90% inhibition of H_2_S production in the presence of cysteine+homocysteine (Fig. 4B).

## Discussion

In this study, we have demonstrated that human CBS reacts with nitrite to generate Fe^II^-NO at rates that are higher than those reported for the hemoglobin T state (*k* = 0.082 M^−1^ s^−1^) and for neuroglobin (*k* = 0.12 M^−1^ s^−1^ and 0.062 M^−1^ s^−1^ for the oxidized and reduced protein). The nitrite concentrations used in these experiments to demonstrate the nitrite reductase activity of human CBS are high, albeit similar to those used previously to measure NO-generation from nitrite by other heme-containing proteins [10], [11]. We note that high K_M_ values (in the millimolar range) for the CBS substrates (e.g. homocysteine) have also been reported consistently by different groups despite the low (micromolar) substrate concentrations present inside cells [54]. Since defects in CBS clearly affect cellular utilization of homocysteine and lead to homocysinuria [18], it raises the possibility that either small molecule or protein modulators in the cell increase the affinity of CBS for its substrates for CBS. However, it is too early to speculate on whether cellular modulation of the affinity of CBS for nitrite occurs.

The in vitro nitrite reductase activity of CBS raises the possibility that it might contribute to NO biogenesis from the nitrite pool particularly under hypoxic conditions, and suggests a possible role for CBS in NO-signaling particularly in tissues where CBS is abundant. The high affinity of hemoglobins for NO begs the question as to how NO can be released efficiently to act as a signaling molecule. In this context, the increase in the dissociation rate constant of NO from Fe^III^ versus Fe^II^-hemoglobin has been proposed as a possible solution, which requires partially oxygenated conditions [55]. The *k*_off_ for NO from the Fe^II^-CBS complex is not known. A rapid rate of dissociation would be advantageous by permitting more facile release of NO from CBS under hypoxic conditions.

A potential role of CBS in NO signaling is supported by the ability of a physiological reducing system to mediate formation of Fe^II^-NO CBS (Fig. 2). Transient formation of Fe^II^-NO CBS could serve as an allosteric switch for CBS. The contribution of CBS to H_2_S is tissue-dependent [56]. Inhibition of CBS under hypoxic conditions when sulfide oxidation is limited, could represent a mechanism for simultaneously decreasing H_2_S production by CBS. The metabolic consequences of CBS inhibition are likely to be complex and would depend on the relative distribution of CBS versus the other H_2_S producing enzymes. Thus, Fe^II^-NO CBS would result in increased homocysteine but decreased cysteine, cystathionine and CBS-derived H_2_S (Fig. 4C). Cystathionine, a product of the canonical serine+homocysteine or the noncanonical cysteine+homocysteine reactions, is cleaved by cystathionase to cysteine. Hence CBS inhibition is predicted to decrease cysteine and H_2_S production by CBS and by the cysteine catabolic pathway, comprising cysteine amino transferase and mercaptopyruvate sulfurtransferase. On the other hand, accumulation of homocysteine would increase the rate of cystathionase-dependent H_2_S formation. However, in tissues in which cystathionase levels are low, e.g. brain [57], formation of Fe^II^-NO CBS is expected to result in homocysteine accumulation and a net decrease in H_2_S synthesis. Similar metabolic changes are predicted for Fe^II^-CO CBS formation.

Both NO and H_2_S are positive effectors of the cardiovascular system and their specific targets are soluble guanylate cyclase and potassium channels, respectively [58]. Our data suggest a molecular mechanism by which CBS might be important for controlling the balance between the NO and H_2_S signaling pathways. Generation of H_2_S and possibly, NO by CBS also suggests a potential role for the enzyme in regulating production of HSNO, a nitrosothiol described as a signaling molecule [59]. Finally, CBS inhibition diminishes intracellular cysteine concentrations in various cell types [60], [61]. Cysteine in turn, is a substrate for H_2_S-generation by cystathionase, a major H_2_S producer, and a limiting substrate for glutathione synthesis (Fig. 4C). Consequently, CBS has the potential to regulate NO, H_2_S and glutathione production either directly or indirectly via its heme-dependent catalytic activity.

## Materials and Methods

### Materials

All reagents were purchased from Sigma unless otherwise specified. Diethylamine NONOate was from Cayman Chemical (Ann Arbor, MI). CBS and MSR were purified as previously described [43], [44].

### UV-visible Spectroscopic Characterization of Nitrite Reduction by Fe^II^-CBS

Sodium dithionite and nitrite stock solutions were prepared under anaerobic conditions. The reaction mixtures containing anaerobic solution of 10 µM CBS and 3 mM dithionite in 0.1 M HEPES, pH 7.4 were incubated in a 500 µl cuvette. Following reduction of Fe^III^-CBS to Fe^II^-CBS, the reactions were initiated by addition of nitrite and its reduction was monitored spectrophotometrically at 37°C. For nitrite-dependence studies, the following nitrite concentrations were used 1, 2.5, 5, 10 and 25 mM. The pH-dependent studies were performed between pH 7.0–8.0, using 0.1 M HEPES buffer at pH 7.0, 7.25, 7.4, 7.75 and 8.0. The low redox potential of the heme iron in CBS makes its reduction by dithionite below pH 7.0 difficult, limiting the pH range for these experiments. For the reduction of nitrite by Fe^II^-CBS generated by the MSR/NADPH reducing system, the reaction mixtures contained 10–15 µM CBS, 5 µM MSR and 1 mM NADPH.

### EPR Spectroscopy of Fe^II^-NO CBS

Samples containing 15–20 µM CBS in 0.1 M HEPES pH 7.4 containing either 3 mM dithionite and 10 mM nitrite or 10 µM MSR and 1 mM NADPH were incubated at 37°C and the formation of Fe^II^-NO CBS product was monitored before transferring the reaction mixtures to sealed EPR tubes. The EPR spectra were recorded on a Bruker ESP 300E spectrometer equipped with an Oxford ITC4 temperature controller. The conditions used for spectral acquisition are described in the figure legend.

### NO Binding to Fe^II^-CBS

Binding of NO to 10 µM CBS in 0.1 M HEPES pH 7.4 was determined using NADPH (200 µM) and MSR (5 µM) as a source of electrons and the NO precursor, diethylamine NONOate (diethylammonium (Z)-1-(N,N-diethyl- NONOate amino) diazen-1-ium-1,2-diolate)) (0–533 µM). Reaction mixtures were prepared in a gas-tight syringe and the NO donor was added last. The syringes were sealed and kept without a headspace to prevent NO escape and the Fe^II^-NO CBS formed was monitored spectroscopically until no further changes were observed.

### Activity Tests on Fe^III^ CBS and Fe^II^-NO CBS Species

The activities of ferric- and ferrous-NO CBS were measured under anaerobic conditions using the radiolabeled assay (using [^14^C]-serine+homocysteine) and the lead acetate assay (using cysteine+homocysteine) as previously described [43], [45].
